# A study on the applicability of the Strengths and Difficulties Questionnaire among low- and higher-educated adolescents

**DOI:** 10.3389/fpsyg.2024.1289158

**Published:** 2024-02-05

**Authors:** Meinou H. C. Theunissen, Marianne S. de Wolff, Iris Eekhout, Coryke van Vulpen, Sijmen A. Reijneveld

**Affiliations:** ^1^TNO Child Health, Leiden, Netherlands; ^2^Pharos, Dutch Centre of Expertise on Health Disparities, Utrecht, Netherlands; ^3^Department of Health Sciences, University Medical Center Groningen, University of Groningen, Groningen, Netherlands

**Keywords:** SDQ, screening, adolescents, education level, community

## Abstract

**Aim:**

The Strengths and Difficulties Questionnaire self-report (SDQ-SR) is a valid instrument for detection of emotional and behavioral problems. The aim of this study was to compare the psychometric properties of the SDQ-SR for low and higher educated adolescents, and to explore its suitability.

**Methods:**

We included 426 adolescents. We compared internal consistency for low-educated, i.e., at maximum pre-vocational secondary education, and higher educated adolescents and assessed whether the five-factor structure of the SDQ holds across educational levels. We also interviewed 24 low-educated adolescents, and 17 professionals.

**Results:**

On most SDQ subscales the low-educated adolescents had more problematic mean scores than the higher educated adolescents. Findings on the invariance factor analyses were inconsistent, with some measures showing a bad fit of the five factor model, and this occurring relatively more for the low-educated adolescents. Professionals and adolescents reported that the SDQ included difficult wordings.

**Discussion:**

Our findings imply that the scale structure of the SDQ-SR is slightly poorer for low educated adolescents. Given this caveat, psychometric properties of the SDQ-SR are generally sufficient for use, regardless of educational level.

## Introduction

About 10% to 25% of adolescents has symptoms of mental health problems, such as low self-esteem, depressive thoughts, and impulsive or maladaptive behaviors (Leaf et al., 1996; Kieling et al., 2011; UNICEF, 2020; The Lancet Regional Health-Europe, 2022). The COVID-19 pandemic has posed additional challenges to the mental health of adolescents by lock down measures and school closures, this may have increased the prevalence of mental health disorders (The Lancet Regional Health-Europe, 2022). These emotional and behavioral problems (EBPs) can negatively impact an adolescent’s development, and can result in lower educational attainment (Veldman et al., 2014), and in persistent serious mental health problems during the life course (Tremblay et al., 2005; Jaspers et al., 2012). Early identification and treatment of EBPs can improve prognosis (Geeraert et al., 2004; Cuijpers et al., 2005; Lavigne et al., 2016).

Community pediatric services play a major role in the early identification of EBPs in children and adolescents. Validated short questionnaires have been shown to improve identification (Theunissen et al., 2019). The Strengths and Difficulties Questionnaire (SDQ) is a short screening instrument widely used to identify EBPs in adolescents, including the support of early identification of EBPs in care settings (Goodman, 1997). The SDQ can be completed by adolescents themselves (aged 11–16) as well as by parents and/or teachers (for children/adolescents aged 4–16). The Self Report (SR) has been shown to be valid as tool for early identification of EBPs in adolescents in a community setting (Goodman, 2001; Muris et al., 2003; van Widenfelt et al., 2003; Van Roy et al., 2008; Kim et al., 2015; Theunissen et al., 2019).

However, an important concern from professionals using the SDQ-SR is that adolescents may not understand the questions correctly, because of the difficulty of many terms used in the SDQ-SR. This can particularly affect responses of low educated adolescents, whose literacy capacities are more limited. Evidence on this issue is lacking, as most studies on the validity of the SDQ-SR have either been performed among higher educated adolescents (Van Roy et al., 2008; Theunissen et al., 2019), or did not differentiate by educational level.

More evidence is needed to confirm that the SDQ-SR measures EBPs in the same way across educational levels. This evidence is even more vital because of the relatively high vulnerability to mental health problems of low educated adolescents (Kaptein et al., 2008; Joffe and Black, 2012). In particular it should be clear whether the SDQ-SR measures the same construct in low and higher educated adolescents. The SDQ consist of a five factor structure: emotional symptoms, conduct problems, hyperactiviy-inattention, peer problems and pro-social behavior (Goodman, 2001). This five factor structure in the SDQ SR has been confirmed in some previous studies in community settings (Koskelainen et al., 2000; van de Looij-Jansen et al., 2011), but not in other studies (Van Roy et al., 2008; Vugteveen et al., 2020). The SDQ should be measurement invariant across educational levels, to guarantee that the SDQ scores of low and higher educated adolescents refer to similar EBPs. Moreover, for optimal use both low educated adolescents and community pediatric professionals should agree on the suitability of the SDQ.

Therefore, aim of the present study was first to compare the psychometric properties of the SDQ-SR (internal consistency and scale structure) between low and higher educated adolescents, and more specifically, whether the five-factor structure of the SDQ-SR is invariant by educational levels. Second, we explored the suitability of the SDQ-SR according to low educated adolescents, and community pediatric professionals.

## Methods

We obtained quantitative and qualitative (interview) data. We also obtained written informed consent for participation in the study from all participating adolescents and from parents of adolescents under the age of 16 years. Ethical approval for this study was granted by the ethics committee of the Heymans Institute for Psychological Research of the University of Groningen in the Netherlands.

### Quantitative study—psychometric properties

For the quantitative study we obtained data about the psychometric properties from adolescents at schools, using two samples.

#### Samples

1. *National sample:* Our first sample included adolescents aged 12–17 years from a national cross-sectional norming study for an intelligence test. Data were gathered in 2016 and 2017 via public schools as part of a norming study for an intelligence test. These schools were located over the whole of the Netherlands equally distributed over rural and urban areas. The sample consisted of 426 adolescents. We included only adolescents with complete data on SDQ-SR and level of education (*n* = 385; 89 low- and 296 higher-educated).
2. *School sample—low educated adolescents:* A second sample included adolescents involved in a newly designed cross-sectional study in three public pre-vocational secondary education schools located in the middle and west of the Netherlands, in 2017 and 2018. The sample consisted of 41 low educated adolescents.

In total, 426 Dutch adolescents (130 low educated and 296 higher educated) participated.

#### Procedure and measures

All adolescents completed the SDQ-SR designed for the 11- to 17-year age range, either at class level or with one-on-one supervision by a test leader. The SDQ-SR consists of 25 items related to children’s strengths and difficulties. Each item is scored on a 3-point scale (0 = not true, 1 = somewhat true, and 2 = certainly true). The SDQ-SR consists of five subscales with each five items: four subscales on difficulties—Emotional symptoms, Conduct problems, Hyperactivity-inattention, and Peer problems,—and one on strengths: Pro-social behavior. An SDQ Total Difficulties Score (TDS) can be calculated by adding up the scores on the first four subscales.

Level of education was determined based on the type of school that adolescents attended and divided into two groups: low (at maximum pre-vocational secondary education) and higher educated adolescents (low general secondary education, higher general secondary education, and pre-university education); 20.9% of all adolescents in the Netherlands attend low-level education (Central Bureau of Statistics, 2021).

#### Analyses

First, we assessed the background characteristics of the sample. Second, we assessed the psychometric properties of the SDQ-SR and compared these between low and higher educated adolescents. We computed the internal consistency (Cronbach’s alphas) of the SDQ-SR (sub)scales (an alpha of >0.70 is considered acceptable) (Streiner, 2003) and the mean values of the SDQ-SR (sub)scales. We tested differences between the two educational levels regarding mean SDQ values using independent sample T-tests. Third, we assessed whether the five-factor structure of the SDQ-SR was invariant across the two education subgroups, by performing multigroup structural equation modeling. Finally, we examined the fit between the scale structure and the observed data for each group (low educated, higher educated, and total group) with confirmatory factor analyses (CFA). The measurement invariance between the groups was evaluated by sequentially testing for invariance in the factor structure (i.e., configural invariance), invariance of the factor loadings (i.e., metric invariance), invariance of the factor intercepts (i.e., scalar invariance), and invariance of the factor intercept means (i.e., residual invariance). Measurement invariance is evaluated by testing the significance of the X^2^ change between two nested models sequentially and allow a CFI change of −0.01 paired with RMSE change of 0.015 (Chen, 2007). The recommended sample size for a CFA is to have at least 100 respondents per group for a multi-group CFA (Kline, 2005). In this study 130 low educated and 296 higher educated completed the SDQ-SR. For the multigroup structural equation and the CFA analyses (single group) we used the following routine measures: root mean square error of approximation (RMSEA) (approximate fit <0.08, and good fit when the RMSEA was <0.05) (Browne and Cudeck, 1993), Standardised Root Mean Residual (SRMR) (good fit for values <0.08), Tucker-Lewis index (TLI), and the Comparative Fit Index (CFI) (TLI and CFI acceptable model fit for values >0.90, and good model fit with values >0.95) (Hu and Bentler, 1999). Items with regression weights <0.30 were considered not to be a fit. Analyses were conducted with SPSS 25 and Lavaan, R Package for Structural Equation Modeling (Rosseel, 2012).

### Qualitative study—opinions regarding suitability of the SDQ-SR

For the qualitative study we obtained data from adolescents and from community pediatric professionals.

#### Samples

1. *Adolescent sample—low educated adolescents:* The sample consisted of 24 adolescents from three pre-vocational secondary education schools.
2. *Professional sample—community pediatric physicians and nurses*: The second sample consisted of 17 nurses and physicians employed at six community pediatric organizations across the Netherlands. They all used the SDQ-SR as part of their initial assessment of emotional and behavioral problems among adolescents.

#### Procedure

Adolescents were interviewed at school by a researcher. They were asked to complete the SDQ-SR out loud. Next, to assess uncertainties we used the method of cognitive debriefing (McColl et al., 2003; García, 2011) whereby adolescents report in their own words what they think to be the meaning of each item of the SDQ-SR. This method is similar to the Teach Back method that is often used in care settings (Yen and Leasure, 2019). At the end, they were asked to mention striking issues and/or difficulties experienced when completing the SDQ-SR. The researcher registered words and items of the SDQ that were perceived as difficult to understand, and reported the adolescents’ general comments about the suitability of the SDQ.

We conducted two (online) focus group interviews with community pediatric professionals (total *n* = 17) about the suitability of the SDQ-SR among adolescents. The interviews were chaired by a researcher. Lead questions related first to the validity of the SDQ-SR, i.e., how often adolescents’ problems were not detected (missed) using the SDQ-SR, or detected falsely. Second, questions related to the strong and weak points of the SDQ-SR. Finally, we addressed the conditions required for successful implementation of the SDQ-SR.

## Results

### Quantitative study—psychometric properties of the SDQ-SR

Table 1 shows the *demographic characteristics* of low and higher educated adolescents. Compared to the low-educated adolescents, the group of higher educated adolescents included relatively more older adolescents (15–17 years) and more girls. However, differences between the two subsamples with regard to adolescent gender and age were small (Cohen effect sizes w: 0.16–0.31).

**Table 1:** Demographic characteristics of participating adolescents, and differences between low and higher educated adolescents.

Characteristic	Low educated	Higher educated	Total
	*N* = 130	*N* = 296	*N* = 426
	*n* (%)	*n* (%)	*n* (%)
Gender			
Boy	80 (61.5)	144 (48.6)	224 (53.7)
Girl	41 (31.5)	152 (51.4)	193 (46.3)
Adolescents’ age (years)			
12–14	82 (63.1)	84 (28.4)	166 (39.0)
15–17	48 (36.9)	212 (71.6)	260 (61.0)
Adolescents’ educational level			
Low (pre-vocational secondary education)	130 (100)		130 (30.5)
Intermediate (lower general secondary education)		77 (26.0)	77 (18.1)
High (higher general secondary education and pre-university)		219 (74.0)	219 (51.4)

Table 2 shows the *Cronbach’s alphas* and the *mean scores* on the SDQ-subscales and TDS for low and higher educated adolescents. The Cronbach’s alphas for the low educated adolescents varied between 0.41 and 0.57, and for the higher educated adolescents between 0.36 and 0.76, with those for total scores being very similar. Comparison of the scores on the SDQ-subscales and TDS in low and higher educated adolescents showed that low educated adolescents had significantly higher mean scores on the TDS and on the subscales Conduct problems, Hyperactivity and Peer problems, i.e., had more problems. For Prosocial we found lower mean scores for low educated adolescents than for higher educated adolescents, i.e., low-educated adolescents had fewer strengths at average.

**Table 2:** Internal consistency, SDQ mean scores and standard deviations (SD) for SDQ subscales in adolescents with low and higher education.

	Cronbach’s ɑ			Mean (SD)			
SDQ scales	Low educated	Higher educated	Total	Low educated	Higher educated	Total	*p*
Total difficulties	0.57	0.58	0.58	10.49 (5.66)	8.49 (4.76)	9.10 (5.13)	<0.01
Emotional symptoms	0.62	0.70	0.68	2.25 (1.99)	2.39 (2.13)	2.35 (2.08)	0.53
Conduct problems	0.52	0.36	0.48	1.96 (1.74)	1.11 (1.12)	1.37 (1.39)	<0.01
Hyperactivity	0.77	0.76	0.76	4.23 (2.58)	3.63 (2.40)	3.81 (2.47)	<0.05
Peer problems	0.41	0.53	0.51	2.05 (1.66)	1.35 (1.49)	1.56 (1.57)	<0.01
Prosocial	0.67	0.60	0.64	7.42 (1.98)	8.17 (1.58)	7.94 (1.75)	<0.01

Table 3 shows the multi-group and single group *invariance indices* of the five factor structure for the two educational groups. Results of the multi-group analyses showed the CFI and TLI to be <0.90, suggesting a bad fit (Table 3). The other goodness-of-fit indices showed adequate fit (RMSEA and SRMR <0.08). We present only the configural model (i.e., with the factor loadings were estimated freely within each education subgroup) because subsequent models impose more restrictions regarding the factor structure, and did not lead to improvement of indices. In other words, the X^2^ changes in the sequential models for measurement invariance, indicated significant worse fit when more parameters were fixed.

**Table 3:** Findings on model fit for the measurement invariance analyses of the SDQ self-report across low and higher educated adolescents.

Model	CFI	TLI	RMSEA	SRMR	SDQ items^a^ *B* < 0.30
Multi group					
Configural model	0.798	0.771	0.057	0.071	
Single group					
Whole group	0.832	0.810	0.052	0.060	11, 22
Low educated	0.779	0.750	0.064	0.085	3, 7, 11, 22
Higher educated	0.808	0.783	0.054	0.064	11, 22

a Table 4 presents all SDQ items.

The single group analyses CFA showed that for all groups (total, low- and higher educated adolescents) the CFI and TLI are <0.90, suggesting a bad fit. The other goodness-of-fit indices showed adequate fit of the five factor model (RMSEA and SRMR <0.08) for all groups. Except for the lower educated group, the RSMSEA approaches 0.08, but was slightly higher (0.085) indicating an inadequate fit. Furthermore, the RMSEA in the whole and higher educated group approaches the 0.05 (0.052 and 0.054, respectively), suggesting a good fit. In sum, the single (total, lower and higher educated groups) and multi-group analyses showed an (almost) acceptable fit for two routine measures of invariance (RMSEA and SRMR) and insufficient fit for two other measures (CFI and TLI). Based on these inconsistent findings, there is a slight indication of measurement variance by educational level, because a bad fit of the five factor model occurs more frequently among lower educated adolescents. However, for both groups variance indices were close to the criterion value.

Two items had regression weights <0.30 (item 11, “I have one good friend or more” and item 22, “I take things that are not mine from home, school or elsewhere”) in all groups. The low educated group also had regression weights <0.30 for item 3, “I get a lot of headaches, stomach aches or sickness” and item 7, “I usually do as I am told”.

### Qualitative study—opinions regarding suitability of the SDQ

We explored the opinions of low educated adolescents and of community pediatric physicians and nurses regarding the suitability of the SDQ-SR. Table 4 shows the difficult to understand words and items of the SDQ-SR as indicated by low educated adolescents and by professionals. According to the adolescents, seven words were particularly difficult to understand. These were Dutch translations of the words/expressions: “restless,” “angry,” “worried,” “down-hearted,” “cheating,” “pick on me” and “taking things that are not mine.” Furthermore, some adolescents reported difficulties with items comprising two sentences, such as items 1, 5, 12, 24, and 25. The general comment was that the Dutch translation of the SDQ-SR is too proper or ‘formal’, resulting in difficult language. The adolescents recommended shortening the number of words in each sentence, and using more understandable words.

**Table 4:** Difficult to understand SDQ words and items as reported by low educated adolescents and community pediatric professionals.

		Adolescents		Professionals
	SDQ items	Word is difficult/total *n*	Item is difficult/total *n*	Difficulties according to professionals
1	I try to be nice to other people. I care about their feelings		3/24	
2	I am *restless*, I cannot stay still for long	11/24	1/24	
3	I get a lot of headaches, stomach aches or sickness		3/24	
4	I usually share with others (food, games, pens etc.)			
5	I get very *angry* and often lose my temper	5/24	3/24	
6	I am usually on my own. I generally play alone or keep to myself		1/24	
7	I usually do as I am told			
8	I *worry* a lot	13/24		x
9	I am helpful if someone is hurt, upset or feeling ill			
10	I am constantly fidgeting or squirming			x
11	I have one good friend or more			x
12	I fight a lot. I can make other people do what I want		8/24	x
13	I am often unhappy, *down-hearted* or tearful	9/24		
14	Other people my age generally like me		1/24	
15	I am easily distracted, I find it difficult to concentrate		2/24	
16	I am nervous in new situations. I easily lose confidence		1/24	
17	I am kind to younger children			
18	I am often accused of lying or *cheating*	7/24		x
19	Other children or young people *pick on me* or bully me	11/24		
20	I often volunteer to help others (parents, teachers, children)			
21	I think before I do things			
22	I *take things that are not mine* from home, school or elsewhere	20/24	1/24	x
23	I get on better with adults than with people my own age			x
24	I have many fears, I am easily scared		3/24	
25	I finish the work I’m doing. My attention is good		3/24	

Professionals reported having perceived the SDQ-SR to be a valid instrument; the outcome of the SDQ-SR often matched their own assessment regarding adolescents’ EBPs. However, they found that the SDQ-SR sometimes falsely identified EBPs. A reason may be that some higher educated adolescents are very critical leading to falsely elevated SDQ-SR scores. Professionals further indicated as weakness of the SDQ-SR that adolescents—regardless of their educational level—experience difficulties with items comprising two sentences. Items found difficult to understand were 8, 10, 11, 12, 18, 22, and 23. Professionals also indicated that the use of the SDQ-SR among adolescents may sometimes lead to socially desirable answers.

A strong point of the SDQ-SR reported by professionals is that it offers a good overall impression of the child’s emotional and behavioral health. More specifically, they mentioned some items to be particularly informative: items 13: ‘I am often unhappy, down-hearted or tearful’, and 19: ‘Other children or young people pick on me or bully me’. However, when asked about the conditions necessary for successful implementation of the SDQ-SR, professionals reported that although the SDQ-SR provides a good overall impression of the child’s EBPs, it includes many outdated and difficult words. They recommended to reformulate the SDQ-SR in simpler language and to provide a training for professionals on using the SDQ, e.g., via an e-learning module.

## Discussion

We found that the internal consistencies of the SDQ-SR subscales were comparable for low and higher educated adolescents, and that mean SDQ TDS scores were higher, i.e., more problematic, for low educated adolescents, as could be expected. For the five-factor structure of the SDQ-SR we found that some measures indicated a slightly invariance by educational level (two out of four measures), while other measures (two out of four) indicated a slightly variance by educational level. The misfit of factor structure occurred relatively more for the low educated adolescents compared to the higher educated or whole group adolescents. Regarding the suitability of the SDQ-SR, professionals perceived the SDQ-SR as a valid instrument, offering a good overview of the child’s wellbeing. However, low educated adolescents and professionals reported that the SDQ-SR includes several hard to understand words. Moreover, they reported difficulties with SDQ-SR items consisting of two sentences.

### Interpretation of the findings

The internal consistency of the SDQ-SR was similar for low and higher educated adolescents but generally relatively poor for some subscales, and for the overall scale. The poor internal consistency of the overall scale contrasts with some previous studies reporting higher Cronbach’s alphas for the SDQ-SR TDS (i.e., 0.75), but is generally consistent with previous studies for some of the other SDQ subscales (Vogels et al., 2009; Theunissen et al., 2013, 2019). Similarly, Vugteveen et al. (2019) found internal consistencies for some SDQ-SR subscales to be insufficient, but to have adequate criterion validity despite. That is rather unexpected as consistency typically is a prerequisite for validity (Moss, 1994). An explanation may be that the various items all measure components relevant for the criterion even though not being related to one unique part. This can then yield a valid subscale even though this not consistently measures one concept.

For the five-factor structure of the SDQ-SR we found some variance by educational level; the scale structure of the SDQ-SR is slightly poorer for low educated adolescents. Nevertheless, the CFA outcomes show that the SDQ-SR performed moderately at conceptual level for both educational groups. Both educational groups have variance indices that are close to the criterion value and the SDQ-SR is therefore sufficient for use regardless educational level. The finding that the SDQ performs moderately at conceptual level is in line with other research (Goodman et al., 2010; Stone et al., 2010; Theunissen et al., 2019). These studies did not differentiate by educational level.

Professionals and low educated adolescents had concerns regarding the suitability of wordings and items of the SDQ-SR, but these findings did not align with our finding of moderate psychometric performance at the conceptual level. More specifically, the items detected by the CFA analysis as having a poor fit were other ones than those mentioned by professionals and adolescents as difficult to understand. An explanation may be that CFA identifies items that do not fit well with the proposed factor structure, which may be rather independent from the difficulty of the wording of an SDQ-SR item. This wording of the items apparently does not have a major influence on the fit with the proposed factor structure.

We further found difficulties in the Dutch translation of the SDQ-SR with some items comprising two sentences and difficult words. Such difficulties are likely to hold for other languages too, and seem to relate partially to outdating of words. For example, the SDQ-SR in English also comprises two sentences such as item 12 “I fight a lot. I can make other people do what I want”. Similarly outdated words are likely to occur in other language-version of the SDQ-SR as this has not been updated in any way since its introduction in 1997, neither in English nor in other languages. The wording of the SDQ-SR thus requires attention, for the Dutch version and also for other languages, in particular with regard to low educated adolescents.

### Strengths and limitations

Our study had several strengths, such as its community-based nature and its use of a combination of quantitative and qualitative data to answer the research questions. However, a limitation may be that we used a relatively small sample, of in total 426 adolescents, in the CFA analyses. However, this still exceeds the recommended minimum sample size for a CFA of 100 respondents per group for a multi-group CFA (Kline, 2005). Another limitation may be that we restricted the interviews to low educated adolescents regarding their perception of the suitability of the SDQ and did not include higher educated adolescents. Higher educated adolescents may have similar difficulties with items comprising two sentences, as indicated by the professionals that participated in this study, so this requires further study.

### Implications

The scale structure of the SDQ-SR is somewhat poorer for low educated adolescents. Given this caveat, psychometric properties of the SDQ-SR are generally sufficient for use among low and higher educational groups. However, the scale structure of the SDQ-SR could be improved, for both educational groups.

Our findings also indicate a need to improve the wording of the SDQ-SR, as clearly expressed by both professionals and adolescents. Adolescents, particularly low educated ones, experience difficulties with the outdated and difficult words, and with items comprising two sentences. This study investigated the experiences difficulties with the wording of the SDQ among professionals and low-educated adolescents. Given the challenges that we found, further research is needed on this issue among higher educated adolescents. These difficulties with the wording of the SDQ are likely to hold for other languages as well, but this deserves further study.

Early detection of EBPs only has value when it is followed by early treatment in improving the prognosis for EBPs. Mental health promotion programmes in schools specifically aim to teach young people social and emotional skills such as self-awareness and resilience. An example of a potentially effective mental health programme in schools is the Social, Personal and Health Education (SPHE) with an aim to promote physical, mental, and emotional wellbeing (Dowling et al., 2019; The Lancet Regional Health-Europe, 2022). The availability and implementation of clinical guidelines are important for providing high-quality primary adolescent health care (Kocken et al., 2022).

## Conclusion

Our study generally yielded reassuring findings regarding the applicability of the SDQ-SR for low educated adolescents compared to higher educated ones. The psychometric properties of the SDQ-SR are sufficient for use among low and higher educated adolescents, but can be improved in both groups by revising SDQ-SR’s wording as well as its scale structure.

## Data availability statement

The raw data supporting the conclusions of this article will be made available by the authors, without undue reservation.

## Ethics statement

The studies involving humans were approved by the Institutional Review Board (or Ethics Committee) of the Heymans Institute for Psychological Research of the University of Groningen in the Netherlands. The studies were conducted in accordance with the local legislation and institutional requirements. Written informed consent for participation in this study was provided by the participants’ legal guardians/next of kin.

## Author contributions

MT: Writing – original draft. MW: Writing – review & editing. IE: Methodology, Writing – review & editing. CV: Writing – review & editing. SR: Writing – original draft.
